# Sickle Cell Anaemia in a Changing World

**DOI:** 10.1371/journal.pmed.1001483

**Published:** 2013-07-16

**Authors:** Edward Fottrell, David Osrin

**Affiliations:** 1 Institute for Global Health, UCL Institute of Child Health, London, United Kingdom; 2 Umeå Centre for Global Health Research, Umeå University, Umeå, Sweden

## Abstract

David Osrin and Edward Fottrell comment on new research by Frédéric Piel and colleagues on the growing burden of sickle cell anemia, and discuss the need for changing policy and health services in response to epidemiologic transitions in child mortality.

*Please see later in the article for the Editors' Summary*

**Linked Research Article**

This Perspective discusses the following new study published in *PLOS Medicine*:

Piel FB, Hay SI, Gupta S, Weatherall DJ, Williams TN (2013) Global Burden of Sickle Cell Anaemia in Children under Five, 2010–2050: Modelling Based on Demographics, Excess Mortality, and Interventions. PLoS Med 10(7): e1001484. doi:10.1371/journal.pmed.1001484

Populations and their health are dynamic. Societal, environmental, and economic changes lead to changes in rates of birth, death, and disease, often described as transitions in mortality, demography, and epidemiology. The notion of epidemiologic transition provides an insight into the relationship between levels of overall mortality and the distribution of its causes [1]–[3], in which the greatest changes arise from the survival of children and young women. Recent falls in global child mortality are good news [4], but will lead to increases in the relative burdens of morbidity and disability in children who would previously have died, and of congenital malformations and inherited disorders. The work of Frédéric Piel and colleagues on sickle cell anaemia (SCA), published in this week's *PLOS Medicine* [5], speaks strongly to this point: SCA is an inherited disease whose global importance will increase in terms of absolute numbers and relative population burden. SCA occurs when individuals are homozygous for sickle haemoglobin (HbS) in place of normal adult haemoglobin, and is the most common form of sickle cell disorder (SCD) [6]. Piel and colleagues have collated HbS allele frequency surveys and used them in models to generate a global distribution map and estimate the numbers of infants born heterozygotic and homozygotic for HbS. Using population and mortality projections, they predict an increase in the numbers of newborns with SCA to over 400,000 in 2050. They also estimate the potential mortality effects of four care-provision scenarios, with a best-case scenario that between 7.5 and 15.5 million newborn lives could be saved, most of them in Africa.

Modelled estimates are a growth area in global health. Whilst useful at supra-national and national levels, their emergence highlights the lack of reliable data on populations, disease, and mortality across most of the world: precisely the sort of information that policy makers and health planners need. The utility of estimates for planning screening programmes, infrastructural and human resource requirements, and clinical care protocols at sub-national levels is likely to be limited where the generalisability of assumptions is challenged by diversity at the local level. Even at a global level, estimates can cause confusion. Research teams using different models may, for example, come to different conclusions [7]. Piel et al. have combined available data, statistical methods, and assumptions to predict current burdens and future trends. Their uncertainties are described clearly, and a preoccupation with methodological critiques can easily distract us from the public health concerns that estimates raise.

The epidemiologic transition has been reframed as a health transition that involves sociocultural, behavioural, and health service factors [8], and policy and health services must respond to changing disease burdens. Unfortunately, the notion of transitions is general. Parallel transitions are happening in different groups within one nation, the best example being differences between socioeconomic groups. Rates of transition vary with local environment, and counter-transition is even possible [9]. Policy makers must set priorities in an environment of multiple burdens, unfinished agendas, competing discourses, and the voices of interest groups [10], a process that has been described as a chaos of purposes and accidents [11]. In an environment of Realpolitik, the generation of estimates of burden is important for advocacy. Characteristically, investigators working in an important public health field that has not received global attention lay down the strategic epidemiology [7],[12], as Piel and colleagues are doing, demonstrating that lack of progress will hinder efforts to attain targets such as those of the Millennium Development Goals.

Quantifying the problem is important, but not sufficient. In a consideration of issue attention for newborn health, Jeremy Shiffman considered four elements: the power of the actors involved, new ideas that can be brought to the table, the characteristics of the issue in terms of attractiveness and tractability, and political context [13]. The kind of strategic epidemiology that the SCA figures exemplify needs to be linked with granular understanding of local epidemiology and service provision [7]. SCA poses a particular challenge in terms of tractability. Haematopoietic stem cell transplantation, an emerging cure, is currently too costly a technology for the countries on which the burden predominantly falls, as is hydroxyurea therapy for children at high risk of illness. Survival, health, and well-being can all be improved substantially, but rely on health care systems with a certain level of functionality. Piel and colleagues suggest that the priority is to identify births of infants with SCA, but that such births could be avoided through genetic counselling and prenatal diagnosis. Termination of pregnancy is one of several options, which include preconception genetic screening and strategic reproductive choices, education for carrier parents, and holistic management from infancy. Quite apart from the logistic and financial challenges, these approaches raise substantial ethical questions summarised in recent work from Ghana [14].

Several interventions would be enormously helpful. Routine newborn screening remains costly—but is likely to become less so—and may miss infants born at home. Penicillin prophylaxis and pneumococcal immunisation are possible in most health care systems. The most beneficial approach involves comprehensive care [15]: family education, routine immunisation, malaria prevention, nutrition and hydration, prophylactic antibiotics, folic acid supplements, transfusion when required, support groups for children and their families, protocols for the management of acute events by health workers and—most importantly—regular follow-up. Human resources for health need to be well trained, and the medicines required need to be affordable and available, including the pain relief required by many people with SCD [16].

Steps towards a systematic approach are being taken [17]. A 2006 World Health Assembly resolution on SCA recommends increased awareness in the international community and emphasises collaboration between countries, including technical support, development of practice models, and coordination [18]. The World Health Organization has published a strategy for the African Region, with targets that include development and implementation of national control programmes in member states with high SCD prevalence, adoption of comprehensive health care management, and establishment of surveillance systems [19]. The estimates from Piel and colleagues underscore the need for both collaborative responses and better data for planning and monitoring.

## Abbreviations

HbS: sickle haemoglobin
SCA: sickle cell anaemia
SCD: sickle cell disorder
